# Influencing factors of obesity in school-age children and adolescents – A systematic review of the literature in the context of obesity monitoring

**DOI:** 10.25646/6729

**Published:** 2020-05-07

**Authors:** Franziska Lehmann, Gianni Varnaccia, Johannes Zeiher, Cornelia Lange, Susanne Jordan

**Affiliations:** 1 Robert Koch Institute, Berlin Department of Epidemiology and Health Monitoring; 2 Formerly Robert Koch Institute, Berlin Department of Epidemiology and Health Monitoring

**Keywords:** SYSTEMATIC REVIEW, OBESITY, CHILDREN AND ADOLESCENTS, PREVENTION, INFLUENCING FACTORS

## Abstract

Around 15% of children and adolescents in Germany are overweight or obese. To support the planning, implementation and evaluation of preventive activities, the Robert Koch Institute (RKI) has developed a population-wide monitoring of influencing factors relevant to the development of obesity during childhood (AdiMon). AdiMon is a web-based indicator system providing population-wide meaningful and regularly updated data on factors that influence obesity in kindergarten-age girls and boys (0- to 6-years-old). Towards the end of 2020, the RKI will expand the indicator system to also cover the 7- to 17-year-old age group. To this end, a systematic review of the literature was conducted, a process which served to identify over 80 relevant factors that influence the development of obesity. These factors have been attributed to the categories behaviour, environment, biology, pre- and postnatal, psychosocial factors and context. Compared to a previous literature review for kindergarten-age children, around one tenth of the influencing factors now identified are new, including ‘peer group influences’ and ‘bullying’. As the results highlight, an array of influencing factors must be considered when expanding the monitoring system, ranging from individual health behaviour to the social framework conditions and environmental factors.

## 1. Introduction

Obesity is considered one of the key public health challenges of our time. Around 15% of children and adolescents in Germany are overweight, approximately 6% are obese [1]. Frequently, obese children and adolescents are also likely to become overweight or obese adults [2]. Being overweight or obese during childhood and adolescence can already cause health problems, with potentially severe health implications later in life [3]. Adult-age obesity is related to metabolic disorders and cardiovascular diseases, certain types of cancer and increased mortality rates [4]. A positive energy balance (taking up more energy than one requires) and biological influencing factors cannot solely explain obesity. Rather, the condition results from a complex interplay between numerous factors [5]. Individual genetic dispositions interact with an environment that has undergone considerable change over the last century and which, in industrialised nations, is characterised for example by a general availability of energy-rich foods. The settings in which individuals live, such as family, school and the community, are themselves influenced by the social, economic and political framework conditions. Key risk and protective factors, such as a health-promoting lifestyle, are acquired during early childhood, meaning that the settings a child grows up in can either reinforce or reduce the influence of such factors. Preventing obesity during childhood and adolescence is therefore of particular importance [6]. To stop the spread of obesity, the World Health Organization (WHO) recommends broad monitoring [7]. Monitoring systems can play a vital role in the planning, implementation and evaluation of prevention measures. Against this backdrop, the Federal Ministry of Health (BMG) has funded the AdiMon project at the Robert Koch Institute (RKI) to develop a population-wide monitoring of factors that influence obesity at childhood age (AdiMon). AdiMon is an web-based indicator system that provides population-wide meaningful and regularly updated data on factors that influence obesity at kindergarten age (0- to 6-years-old) [8, 9]. To describe the diverse set of factors that influence obesity, numerous sources of data were used, and meaningful indicators were developed. These were put into scientific context and published in concise fact sheets.

Beyond kindergarten, school age is a further key stage for the prevention of obesity. The prevalence of obesity among school-age boys and girls increases significantly. While 3.2% of 3- to 6-year old girls and 1.0% of boys are obese, for 14- to 17-year-old adolescents the figures are 7.7% (girls) and 9.2% (boys) [1]. Yet it is not only the prevalences of obesity that increase at school age; children and adolescents who are obese at school age are also more frequently obese as adults than children who are obese in kindergarten [10, 11]. School entry age is therefore considered a critical moment for the manifestation of obesity among children and adolescents. Against this backdrop, it is important to focus on the causes and spread of obesity among girls and boys at school age. By the end of 2020, we will therefore have expanded the indicator system to also cover the 7- to 17-year-old group. For this age group, the school setting is a key factor whose importance is increasing with the spread of all-day schools [12]. Entering school leads to great changes in the lives of children. With primary school, children’s everyday family routines become fundamentally restructured and demands on children increase, e. g. the need to be punctual and sit all day [13]. Furthermore, adolescence is a life phase characterised by numerous changes and challenges. During the biological process of development, the puberty, adolescent’s bodies undergo physiological and hormonal changes that they must confront. At the psychosocial level, the step from childhood to adolescence is characterised by the development of a more individual personality structure and people’s ever greater independence in their social environment [13]. For adolescents, the role played by peers regarding health behaviour increases in a phase where they are becoming ever more independent of their parents [14, 15]. To take account of this special phase in life, and to expand the indicator system, a systematic review of the literature to detect relevant influencing factors for obesity in school-age girls and boys was conducted. In the following, we present and discuss the methodological approach that was applied in the literature review, as well as the results. The latter provided us with a basis to expand the current monitoring system, because so far systematically compiled data on the causes and levels of obesity among school-age girls and boys in Germany had not been available.

## 2. Methodology

This systematic review of the literature was conducted in December 2018 in the Scopus, PubMed and Embase databases, the Web of Science and the Cochrane Library. Systematic reviews published between 2006 and 2018 were taken into account. The search string was built on the terminological categories influencing factor (determinants, causes, correlates, predictors, factors, origins, etiology, and understanding), obesity (obesity, adiposity) and target group (child, youth, adolescent). By using Boolean operators, keywords and wildcards, a database conforming combination of the three categories was created, whereby searches were limited to the titles and abstracts of publications.

After removing duplicates, the titles and abstracts were reviewed and the full texts read. Articles were excluded which were (1) not systematic reviews; (2) conference contributions; or (3) systematic reviews on measures and interventions. Further exclusion criteria were (4) no relevance of the influencing factors in Germany (for example an analysis of the role played by socioeconomic status in developing countries); (5) no relevance of an influencing factor for the population as a whole (such as rare genetic diseases); (6) lacking relevance of an influencing factor for the 7- to 17-year-old age group; and (7) no obesity relevant outcome. Furthermore, when (8) articles were published in non-covered languages (all languages except for German, English, Spanish and Portuguese), or (9) when the full text of an article was not available, the corresponding publications were also excluded.

The influencing factors identified in the reviews were inserted into a table matrix and differentiated by influencing area. The entire process was conducted as a dual control process. The literature was reviewed and influencing factors identified independently by two researchers. When cross-checking resulted in differences in the identified influencing factors, these were discussed together with a third researcher until consensus was achieved.

**Figure 1:**
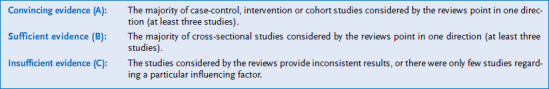
Evaluation of evidence Source: Own figure

For the identified influencing factors, the following step consisted in evaluating the evidence provided. Information on the methodology applied and the results of the studies considered by the reviews was therefore gathered (Figure 1). Three levels of evidence were distinguished: convincing, sufficient and insufficient. Influencing factors were excluded if in the majority of studies on which reviews were based (at least three studies), no indication could be provided for a relationship with the development of obesity during childhood and adolescence. Influencing factors with only insufficient evidence were nonetheless included to take into account more recent research results with fewer studies. The influencing factors identified by the literature review were discussed during a workshop in May 2019 with external experts from public health practice, politics and science, and consolidated and evaluated with regard to their relevance for the prevention of obesity.

While the AdiMon system of indicators does consider measures and interventions relevant to the field of obesity, for methodological reasons these were not part of this literature review, because reviews on influencing factors and interventions appeared to require different search strategies. Correspondingly, over the course of the project, a further literature review will therefore be conducted. It will provide information on the scientific evidence regarding measures and interventions relevant to obesity at the individual, setting and population level.

## 3. Results

**Figure 2:**
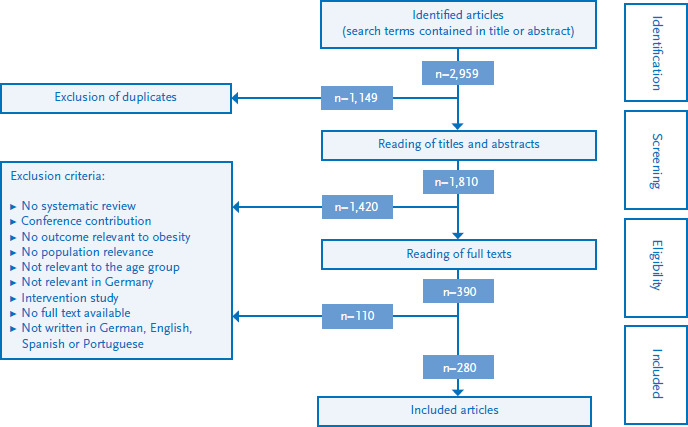
Flow diagram on the systematic literature review Source: Own diagram

The described literature review provided 2,959 hits, of which 280 fulfilled our inclusion criteria (Figure 2). A total of 80 influencing factors that are relevant to the development of obesity in school-age girls and boys in Germany were identified. Around one tenth of these are factors that were not identified by the AdiMon project review conducted for kindergarten-age children [9].

**Figure 3:**
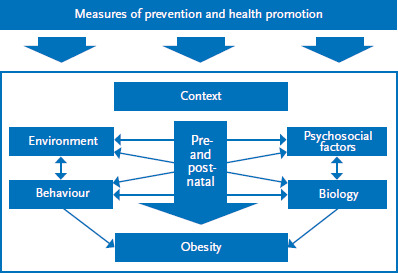
Simplified cause-effect model of obesity at child and adolescent age Source: Modified according to Varnaccia et al. 2017 [8]

The detected influencing factors were categorised according to the areas behaviour, environment, biology, pre- and postnatal, psychosocial factors and context shown in the simplified cause and effect model (Figure 3). The model was developed during the initial phases of obesity monitoring for kindergarten-age children [8]. It shows that behavioural (such as physical activity) and biological factors (such as genetic disposition) are relevant for the development of obesity. Also prenatal factors (such as weight gain by mothers during pregnancy) and factors during early childhood (such as breastfeeding) influence the development of obesity. Furthermore, psychosocial factors (such as how parents perceive the weight of their children) can impact the development of obesity during childhood and adolescence. Environmental factors are mostly related to the settings a child grows up in, such as family, neighbourhood or kindergarten (for example the food offered there). The mentioned influencing factors are themselves influenced by contextual factors. These include cultural and sociodemographic factors (such as poverty). Moreover, prevention and health promotion measures can influence the levels of childhood obesity. We will now discuss these areas in more detail and describe them in correlation to the results of some identified reviews. An overview of all the influencing factors identified in the review of the literature is included in the annex (Annex Table 1).

### 3.1 Behaviour

#### Nutrition

A positive energy balance, which occurs when caloric intake exceeds energy expenditure, is considered the central cause of obesity [16]. Consuming high-energy-dense foods and meals can favour a positive energy balance and promote obesity [17]. It is assumed that when consuming high-energy-dense meals compared to low-energy-dense meals, more energy is taken up by the body before satiety is felt [17]. Concerning nutrient intake, consuming large amounts of simple carbohydrates, proteins and fats are being discussed as factors that could encourage obesity, as opposed to consuming high amounts of dietary fibre, which it is presumed to reduce a person’s likelihood of becoming obese [18–21]. Numerous longitudinal studies have related the regular consumption of sugar-sweetened beverages and fast food to the development of obesity in children and adolescents [22, 23]. Regularly eating fruit and vegetables are considered factors that protect against becoming obese, even though the results of longitudinal studies for children and adolescents are not entirely consistent [24].

#### Physical activity, sedentary lifestyle and sleep

As it increases energy expenditure and contributes to a more even energy balance, physical activity is considered to be a factor protecting children and adolescents from becoming obese. Several cross-sectional and longitudinal studies have shown an inverse relationship between physical activity and childhood obesity [25, 26]. A sedentary lifestyle and in particular using screen-based media can promote obesity [27]. In addition to the reduced energy requirements of people with a sedentary lifestyle, the effects of advertisements for particular foods (such as sweets) and the consumption of energy-rich foods while using screen-based media are being discussed as potential causes for an increased obesity risk [28]. Studies concerning the 7- to 17-year-old population are, however, not consistent. Too little sleep is a further obesity risk factor currently being discussed, a habit that can cause alterations to the metabolism and at the hormonal level that favour obesity. Longitudinal study results support this hypothesis [29].

### 3.2 Environments

#### Living environments

The living environments of children and adolescents play an important role in the development of obesity. We use the term living environment here in the sense of a definable social system to describe the framework conditions in which people live, learn, work and consume [30]. Relevant living environments for school-age girls and boys are the family and home, school and their immediate neighbourhoods (such as the municipality in which they live). Parent weight and health behaviour are principal influencing factors of the family. A meta-analysis indicates that children whose parents are obese have a significantly higher obesity risk [31]. This relationship was stronger for older than for younger children, as well as when both parents were obese. Parent weight has a strong genetic and epigenetic component, which means there are overlaps with biological factors. A number of correlation studies provide insights into how parental health behaviour, such as dietary habits and levels of physical activity, play out in children [32]. Moreover, a review relying principally on cross-sectional studies indicated that shared family meals are associated with a lower obesity risk [33]. For the school environment, a negative influence of unhealthy food options on the weight of adolescents is being discussed; however, only very few studies to date have confirmed such a correlation [34]. For the living environment municipality, the availability of spaces that encourage physical activity [35], security of residential areas [36] and shopping and food options that promote a healthy diet [35] are considered to be factors that protect against obesity. However, the results in the identified reviews, which are mostly based on cross-sectional studies, are inconsistent.

#### Economic factors

Economic factors that are being discussed as influencing obesity in school-age children and adolescents include the cost of participating in physical activities as well as the price of food and meals. The few available studies show that accessible prices for exercise opportunities can help increase levels of physical activity, as well as that increases to the price of fruit and vegetables are associated with gaining weight [37, 38]. In addition, cross-sectional and longitudinal studies show that advertisements for high-energy-dense foods (TV ads for sweets or campaigns sponsored by the producers of foods that promote obesity) can increase the consumption of such foods and therefore promote obesity [39].

### 3.3 Biological factors

Numerous longitudinal studies have shown that genetic predispositions increase the obesity risk of children and adolescents [40, 41]. Furthermore, certain hormones (such as leptin [42]) and the composition of a person’s intestinal flora [43] are being discussed as relevant influencing factors for obesity. Leptin plays a role in managing hunger and satiety and is fundamental to the energy balance. Raised serum leptin levels have been observed in obese children [42]. A number of mechanisms are being discussed to explain the influence the gut microbiota has, such as gaining energy from indigestible carbohydrates as an additional source of energy [43]. Further potentially relevant biological influencing factors under discussion include certain diseases (such as asthma [44]), virus infections (for example adenovirus infections [45]), as well as the side-effects of certain medications (such as antibiotics [46]). Furthermore, levels of physical fitness have also been related to developing obesity. Several longitudinal studies show that high cardio-respiratory fitness (endurance), good motor capacity and pronounced muscular fitness can prevent obesity in children and adolescents [47–49]. Compared to physical activity, which describes a specific behaviour, physical fitness is a physical trait and therefore related to biology.

### 3.4 Pre- and postnatal

#### Pregnancy

Maternal obesity in the early stages of pregnancy increases the likelihood of their children being obese during childhood and adolescence. Numerous prospective studies provide consistent results for this hypothesis [50, 51]. In addition, several longitudinal show that high maternal weight gain during pregnancy and (gestational) diabetes are risk factors for the development of obesity during childhood and adolescence [51, 52]. The same holds true for mothers who smoke during pregnancy [53, 54].

#### Early childhood

The results of systematic reviews that consider a high birth weight and rapid weight gain after birth are largely consistent. The results show that these factors can promote obesity at child and adolescent age [55, 56]. Breastfeeding, to the contrary, appears to be a protective factor. As numerous longitudinal studies have shown, how long and how (e.g. exclusive breastfeeding or breastfeeding and complementary feeding) a baby is breastfed potentially influence obesity risks [57, 58].

### 3.5 Psychosocial factors

#### Children and adolescents

Certain psychosocial factors are being discussed as potentially relevant for child and adolescent obesity. Various long-term studies indicate depression could promote obesity among girls and boys [59]. It is assumed this association is bi-directional, i.e. obesity would then also be a risk factor for depression. Factors that could encourage obesity in the context of a manifest depression and that are currently being discussed include changes to appetite and dietary habits, such as developing a preference for carbohydrate-rich foods, as well as more sedentary habits and sleeping disorders [60]. A number of cross-sectional studies have highlighted a connection between stressful (traumatic) life events, such as suffering violence, and a greater risk of becoming obese [61, 62]. High levels of stress, eating disorders (such as binge eating) and suffering bullying are further influencing factors that encourage obesity and have been described in the literature [60–63]. Longitudinal studies have also shown a weak connection between low self-esteem and the risk of becoming overweight or obese [59]. Overall, the evidence for the stated psychosocial influencing factors has to be rated insufficient and there is a lack of models to explain relationships and effect mechanisms.

#### Parents and the peer group

Parents facing high levels of stress and mothers suffering from depression can increase a child’s obesity risk [64, 65]. How parents perceive a child’s weight can also be a relevant factor in whether a child becomes obese or not. It is assumed that parents who are conscious that their children are overweight will take action to prevent them from becoming obese [66]. In addition, low levels of parental health literacy could encourage childhood obesity [67]. Moreover, for adolescents, the influence of their peer group can lead to similar weight developments and obesity related behaviours [68]. To date, these influencing factors have only been insufficiently studied.

### 3.6 Context

Longitudinal studies indicate that in high income countries such as Germany children and adolescents of low socioeconomic status are more often overweight or obese than their peers from high status groups [69]. In particular, a low parental educational level is considered a risk factor for children and adolescents with regard to the development of obesity, and this factor plays out more heavily in younger than older children. [70]. It is assumed that a low parental educational level is often associated with little health knowledge and only insufficient health literacy, making a health-promoting lifestyle (for example a balanced diet) less probable and therefore supporting the development of obesity in children [70]. Longitudinal studies underline that a low household income can be a risk factor for the development of obesity in children and adolescents, as limited financial resources make it more difficult to access leisure-time activities that involve physical activity and to pay for a healthy diet [71].

In cross-sectional studies it has been observed that children and adolescents with a migration background are more frequently overweight or obese than adolescents with no migration background. Differences in diet and physical activity are thereby mentioned as possible explanations [72]. Importantly, however, the influence a migration background has on becoming obese depends on a number of different factors and having a migration background in Germany is often related to further factors that can encourage obesity (for example a lack of financial resources) [73].

## 4. Discussion

This article aims to provide an up-to-date overview of the factors influencing obesity in school-age girls and boys and which are the basis for the indicators of a population-wide monitoring system. As the results of the literature review emphasise, the causes of obesity in school-age girls and boys are diverse and not limited to factors of individual behaviour. Comparisons with the established explanatory models for obesity at child and adolescent age also highlight this [74, 75]. Between the identified factors, it is presumed that links and diverse interactions at numerous levels exist [76]. In addition, the different areas play out differently in the development of obesity regarding their direct or indirect influence (Figure 3). Based on this literature review, it is not possible to quantify the effects of particular factors, as other studies have attempted to do [77–79]. However, the diversity of the influencing factors that were identified highlights that tackling obesity in childhood and adolescence calls for measures in more than just one area. Rather, the strategy should aim to develop health promoting framework conditions in living environments, as this would encourage positive health behaviour [80]. However, this depends on equally addressing the political and economic framework conditions. Monitoring thereby plays a key role: it ensures that developments over time can be tracked and needs to take action can be recognised at an early stage, thereby providing important insights for the planning, implementation and evaluation of prevention measures and strategies [81].

Systematic literature reviews have their limitations. We have accounted for publications with the outcome obesity, but also with a number of alternative outcomes, such as changes of other anthropometric measures (such as body mass index) or behaviours relevant to obesity (such as physical activity). Restricting ourselves to systematic reviews and a search string that looks at titles and the abstract of publications are further limitations. There were also certain limitations with regard to the age groups. The age groups described in the literature often did not fit precisely the 7- to 17-year-old group and only considered a subgroup or also included younger and/or older people. To simplify matters, the terms child and adolescent were therefore used in the results section. It is also important to mention that substantial discrepancies existed in the number of studies found for each factor. The majority of the identified publications dealt with behaviour-related and biological influencing factors. For the areas environment, context, psychosocial factors, as well as pre- and postnatal factors, the search provided fewer systematic reviews. The differing number of identified reviews for specific influence fields possibly also influenced the evaluation of evidence. The synopsis of influencing factors included all factors associated with obesity. These include findings from cross-sectional studies, which do not allow a clean separation of cause and effect and are therefore less robust. This means that in some cases it remains unclear whether the observed relationships are causal, as well as which side of this causality is the effect and how the mechanisms underlying these effects are structured. In addition, the influencing factors which have been considered as only insufficiently researched should nonetheless be considered in the population-wide monitoring. Mostly these are from the areas environment and psychosocial factors, and were included to account for a broad spectrum of influencing factors. To prevent distortions in the compilation of influencing factors and the evaluation of supporting evidence, the results of the literature review were consolidated during a workshop with external experts.

## Conclusion

The results of our systematic review of the literature highlight the complex structure of influencing factors that underlie obesity in school-age girls and boys. Compared to an earlier review on factors that influence the obesity of 0- to 6-year-old children, new influencing factors were identified for the 7- to 17-year-old group. These include aspects from the school environment and factors that gain in importance at secondary school age, such as the influence of the peer group or having suffered bullying.

The literature review also showed that over the last years the causes of obesity among children and adolescents have been amply researched. Further research is nonetheless required. For environmental and psychosocial influencing factors, for example, only few studies are so far available. More findings are required that would allow us to assess the relevance of particular influencing factors for the development of obesity, explain the underlying effect mechanisms and provide effective settings-based prevention measures. Furthermore, future concepts of obesity prevention will need to be based within a broader context. This could include considering the development of indicators to account for the interactions between obesity prevention and environmental and climate aspects [82].

The next step of the AdiMon project will involve reaching a consensus between experts on robust indicators for specific influencing factors, and acquiring the corresponding data sources. Until October 2020, the new indicators for school-age children and adolescents will be integrated into the existing system of indicators and published on the AdiMon website. In the medium to long term, AdiMon will be updated regularly and thereby allow us to map the changes of the diverse influencing factors. AdiMon will thereby provide starting points for new and adequate prevention strategies that will target the individual-, setting- and population-level.

## Key statements

- The systematic literature review provided over 80 factors relevant to the development of obesity in school-age girls and boys.
- Obesity results from a complex interplay between numerous influencing factors from a number of fields.
- Compared to a previous literature review for kindergarten-age children conducted in the context of the AdiMon project, the number of identified influencing factors has increased by around one tenth.
- The identified reviews mostly focused on behavioural and biological influencing factors.
- Environmental and psychosocial influencing factors were only examined by a small number of systematic reviews.

## Appendix

**Annex Table 1:** Influencing factors of obesity in school-age girls and boys (updated: May 2019) Source: Own table

Influencing factor	Relationship	Literature*
**Behaviour**		
**Physical activity**		
Physical activity	Regular physical activity can help prevent obesity.	[1, 2]
Sports activity	Regular sports activity can help prevent obesity	[3, 4]
**Sedentary behaviour**		
Sitting time	Regularly spending time sitting can favour obesity.	[5, 6]
Screen time	Regularly spending time in front of a screen can favour obesity.	[5, 7]
**Diet**		
Energy intake	Taking up a high amount of energy can favour obesity.	[8]
Carbohydrates	Taking up large amounts of carbohydrates and/or consuming particular types of carbohydrates such as fructose, can favour obesity.	[9–11]
Proteins	Taking up large amounts of proteins in early childhood can favour obesity.	[12]
Fat	Taking up large amounts of fat can favour obesity.	[13]
Fibres	Taking up large amounts of fibres can help prevent obesity.	[14]
Vitamins and minerals	Vitamin and mineral uptake can be a relevant factor for obesity.	[15–17]
Energy density	High-energy-density foods can favour obesity.	[18]
Sugar-sweetened beverages	Regularly consuming sugar-sweetened beverages can favour obesity.	[19]
Fruit and vegetables	Regularly eating fruit and vegetables can prevent obesity.	[20]
Fast food	Regularly eating fast food can favour obesity.	[21, 22]
Milk and milk products	Regularly consuming milk and milk products can help prevent obesity.	[23]
Dietary patterns	Particular dietary patterns (such as eating mainly highly processed foods) can be a relevant factor for obesity.	[24]
Meal frequency	A high frequency of meals can help prevent obesity.	[25]
Breakfast	Regularly eating breakfast can help prevent obesity.	[26]
**Sleep**		
Sleep duration	A short sleep duration can favour obesity.	[27–29]
**Environment**		
**Living environment**		
Parental obesity	Parental obesity can favour obesity in their children.	[30]
Parental health behaviour	A healthy parental lifestyle can help prevent obesity in their children.	[31]
Sibling health behaviour	A healthy lifestyle of siblings can help prevent obesity.	[31]
Home food environment	A balanced offer of food and meals at home can help prevent obesity.	[31]
Shared family meals	Shared family meals can help prevent obesity.	[32]
School food environment	A balanced offer of food and meals at school can help prevent obesity.	[33]
Opportunities for physical activity at school	Spaces and opportunities for physical activity at school can help prevent obesity.	[34]
Spaces for physical activity in the neighbourhood	Spaces for physical activity in the neighbourhood (such as parks, sports facilities, playground) can help prevent obesity.	[35]
Sports offers	Sports offers (for example sports clubs) can help prevent obesity.	[34]
Activity-promoting infrastructure	Activity-promoting infrastructure (for example walkability) can help prevent obesity.	[35]
Security in the neighbourhood	A secure environment in the neighbourhood or one that is perceived as being secure can help prevent obesity.	[36]
Shops and food options	Shops and food options (for example fast food restaurants) can be a relevant factor for obesity.	[35]
Degree of urbanisation	Growing up in rural communities can favour obesity.	[37]
Regional deprivation	Growing up in a deprived area can favour obesity.	[38]
**Economic factors**		
Costs related to physical activities	Costs related to physical activities can be a relevant factor for obesity.	[34]
Prices of food and meals	The price of food and meals can be a relevant factor for obesity.	[39]
Advertisements	Advertisements for high-energy-density foods can favour obesity.	[40]
**Other factors**		
Pollutants	Certain pollutants (such as bisphenol A) can favour obesity.	[41]
Portion sizes	Large portion sizes can favour obesity.	[42]
**Biology**		
**Genetic factors**		
Genes and gene combinations	Certain genes and combinations of genes can be relevant factors for obesity.	[43]
**Hormonal factors**		
Hormones	Certain hormones (such as leptin) can be a relevant factor for obesity.	[44]
**Micro-biological factors**		
Intestinal flora	Intestinal flora composition can be a relevant factor for obesity.	[45]
**Physical fitness**		
Motor fitness	Strong motor fitness can help prevent obesity.	[46]
Cardio-respiratory fitness	Strong cardio-respiratory fitness can help prevent obesity.	[47]
Muscular fitness	Strong muscular fitness can help prevent obesity.	[48]
**Other factors**		
Puberty	Precocious puberty can favour obesity.	[49]
Diseases and viruses	Certain diseases (such as autism and asthma) and viruses (such as adenoviruses) can favour obesity.	[50–52]
Medication	Certain medications (such as antibiotics) can favour obesity.	[53, 54]
**Pre and postnatal**		
**Pregnancy**		
Maternal body mass index	A high maternal body mass index in the early stages of pregnancy can favour childhood obesity.	[55, 56]
Maternal weight gain	A high maternal weight gain during pregnancy can favour childhood obesity.	[55, 57]
Maternal diabetes	Maternal (gestational) diabetes during pregnancy can favour childhood obesity.	[55, 58]
Maternal smoking	Maternal smoking during pregnancy can favour childhood obesity.	[59, 60]
Maternal passive smoking	Maternal passive smoking during pregnancy can favour childhood obesity.	[59, 61]
**Early childhood**		
Weight at birth	A high birth weight can favour obesity.	[55, 62]
Caesarean section	Caesarean section can favour obesity.	[63]
Breastfeeding	Breastfeeding can help prevent obesity.	[64, 65]
Duration of breastfeeding	Breastfeeding over an extended period can help prevent obesity.	[64, 65]
Form of breastfeeding	The form of breastfeeding (for example any form of breastfeeding, exclusive breastfeeding, bottle feeding) can be a relevant factor for obesity.	[55]
Complementary feeding	Early complementary feeding (before the fourth month) can favour obesity.	[66]
Rapid weight gain	A rapid weight gain during the first months of life can favour obesity.	[67]
Tonsillectomy	A tonsillectomy can favour obesity.	[68]
Child-care attendance	Attending child-care at an early age (0- to 2-years) can favour obesity.	[55]
**Psychosocial factors**		
**Children and adolescents**		
Personality traits	Certain personality traits (such as impulsivity, self-regulation) can be relevant factors for obesity.	[69]
Self-esteem	Having low self-esteem can favour obesity.	[70]
Family climate	An unfavourable family climate can favour obesity.	[71]
Parent-child relationship	A weak parent-child relationship can favour obesity.	[72]
Social support	Social support can help prevent obesity.	[73]
Stress	High levels of stress can favour obesity.	[74]
Stressful life events	Stressful life events (such as maltreatment) can favour obesity.	[75, 76]
Bullying	Bullying can favour obesity.	[76]
Eating disorders	Eating disorders (such as restrictive eating and binge eating) can favour obesity.	[77]
ADHD	Attention Deficit and Hyperactivity Disorder (ADHD) can favour obesity.	[78]
Depression	Depression can favour obesity.	[79]
**Peer group**		
Peer group	The peer group can be relevant for obesity.	[80, 81]
**Parents**		
Stress levels	High levels of parental stress can favour obesity in their children.	[82]
Depression	Maternal depression can favour childhood obesity.	[83]
Parental perception of child’s body weight	Parental misperception of their child’s body weight can favour childhood obesity.	[84, 85]
Health literacy	Insufficient parental health literacy can favour obesity in their children.	[86]
Parenting style	An authoritative parenting style can help prevent obesity.	[87, 88]
Feeding style	How and when a child is given food can be relevant for obesity.	[87]
**Context**		
**Sociodemographic factors**		
Social status	A low social status can favour obesity.	[89, 90]
Child’s educational level	A low child’s educational level can favour obesity.	[91]
Parental educational status	A low parental educational status can favour obesity in their children.	[91]
Parent employment	The numbers of hours parents work can be relevant for obesity in their children.	[89]
Poverty	Financial poverty can favour obesity.	[92]
Migration background	A migration background can be relevant for obesity.	[93]
Single parent families	Growing up in a single-parent household can favour obesity.	[31]
Siblings	Growing up as an only child can favour obesity.	[94]
Order of birth	The order of birth can be relevant for obesity.	[94]

^*^ The references represent a selected number of publications (in the review of the literature 280 relevant publications were identified).
